# Efficacy and safety of ozone therapy for knee osteoarthritis: an umbrella review of systematic reviews

**DOI:** 10.3389/fphys.2024.1348028

**Published:** 2024-02-20

**Authors:** Valéria T. S. Lino, Daniel S. Marinho, Nadia C. P. Rodrigues, Carlos A. F. Andrade

**Affiliations:** ^1^ Primary Care Department- Germano Sinval Faria School Health Center, Sergio Arouca National School of Public Health- Oswaldo Cruz Foundation, Rio de Janeiro, Brazil; ^2^ Department of Epidemiology and Quantitative Methods in Health, Sergio Arouca National School of Public Health- Oswaldo Cruz Foundation- Rio de Janeiro, Rio de Janeiro, Brazil; ^3^ Institute of Social Medicine, State University of Rio de Janeiro, Rio de Janeiro, Brazil; ^4^ Faculty of Medicine, Vassouras University–Vassouras–Rio de Janeiro, Vassouras, Brazil

**Keywords:** ozone therapy, knee osteoarthritis, umbrella review, systematic review, AMSTAR2, randomized controlled trials

## Abstract

The objective of this study is to evaluate the effectiveness and safety of ozone therapy (OT) in the treatment of knee osteoarthritis (KOA), which is the most common form of the disease. We analysed systematic reviews (SRs) of randomised controlled trials (RCTs) using the “A MeaSurement Tool to Assess systematic Reviews” (AMSTAR2) instrument to evaluate their quality. We developed a narrative synthesis report with eight SRs (15 RCTs/3,685 patients) to summarise the findings. The AMSTAR2 analysis indicated that all reviews had critically low confidence ratings. Statistically significant effects in pain reduction using OT compared to placebo groups were reported in three SRs. OT was shown to be comparable to other therapies in one SR and not superior in the other five. Six SRs highlighted the need for additional RCTs with improved methodological quality to confirm the efficacy of OT for KOA. SRs found fewer consistent effects for improving joint function. Regarding safety, seven SRs reported a low prevalence of minor adverse events linked with OT. Finally, this umbrella review highlights the beneficial effects and safety of OT in the treatment of KOA, particularly in pain control. The low methodological quality of RCTs and SRs limits the possibility of drawing conclusions on the effectiveness of the procedure in comparison to other therapies. Ensure adequate compliance with guidelines such as Preferred Reporting Items for Systematic Reviews and Meta-Analyses (PRISMA) and AMSTAR2 has the ability to improve the quality of SRs in this area.

## Introduction

Osteoarthritis (OA) is the most common form of arthritis and represents an important public health problem, with an increase in years lived with disability in most countries. This burden is mainly associated with population aging and obesity. Knee osteoarthritis (KOA) is the most frequent presentation of OA (Safiri et al., 2020).

OA management emphasizes non-pharmacological interventions as the first line of treatment, encompassing education, self-management, exercise, and weight loss for overweight or obese individuals, along with the judicious use of walking aids when appropriate. Exercise therapy stands out as a pivotal component in effectively mitigating pain and enhancing joint mobility in OA patients. Evidence supports the efficacy of weight loss interventions, but combining dietary management with exercise yields superior outcomes in terms of pain reduction and functional improvement compared to either approach in isolation (Hunter and Bierma-Zeinstra, 2019).

Although corticosteroids and hyaluronic acid intra-articular (IA) injections are available treatments, their outcomes are subject to controversy. Hence, considering the non-steroidal anti-inflammatory drugs, adverse reactions limit the long-term utilization of these drugs (Xing et al., 2017; Hunter and Bierma-Zeinstra, 2019).

In advanced KOA cases, total knee arthroplasty emerges as the most effective treatment option. However, the procedure is not without risks of complications, necessitating a thorough assessment of the patient’s condition and a careful consideration of alternative treatments before proceeding with surgery. In addition, pain after surgery persists in 20% of people. This leads to impaired health-related quality of life, emotional stress, depression, and social isolation (Wylde et al., 2018). Thus, therapeutic alternatives that slow the progress of KOA may reduce the disease burden.

Oxidative stress and chronic inflammation are associated with joint degeneration and pain in OA. Injured chondrocytes release pro-inflammatory cytokines and damage their own DNA. Ozone therapy (OT) reduces inflammation and oxidative stress that induces organ damage in chronic diseases (Davies et al., 2008; de Sire et al., 2022; Sconza et al., 2023). Like physical exercise, ozone induces low–moderate level oxidative stress and triggers a series of intracellular metabolic processes that improve cell functionality. Thus, repeated exposure to OT may cause resistance against oxidative stress (Bocci and Valacchi, 2015; de Sire et al., 2022; Serra et al., 2023). In a similar pattern, intra-articular ozone is able to induce the generation of reactive species of oxygen (ROS) and lipid oxygen products (LOPs), stimulating the antioxidant system and creating an environment that counteracts the proinflammatory and pro-oxidative circuits present in knee OA (Davies et al., 2008; Borrelli et al., 2015; Sconza et al., 2023). In this scenario, OT becomes a low-cost therapeutic candidate for the management of KOA.

Clinical trials indicate varying outcomes of OT on pain and joint function in KOA. This variability may stem from differences in treatment protocols and administration methods, such as variations in the injected ozone’s volume and concentration, the number of therapy sessions, and the choice of substances used as a control. These disparities highlight the need for standardization and consistency in research methodologies to better understand the true impact of OT on KOA (Giombini et al., 2016; Duymus et al., 2017; Lopes de Jesus et al., 2017; Babaei-Ghazani et al., 2018; de Sire et al., 2022; Aliyev et al., 2023; Sconza et al., 2023) The intervention seems to be a safe approach (Hashemi et al., 2015; Chansoria et al., 2016; Lopes de Jesus et al., 2017; de Sire et al., 2022; Serra et al., 2023). Several systematic reviews (SRs) summarize the available evidence on the efficacy of OT in KOA pain and joint function, but the lack of a standardized protocol limits us from drawing conclusions on the efficacy of OT (Raeissadat et al., 2018a; Costa et al., 2018; Li et al., 2018; Arias-Vázquez et al., 2019a; Oliviero et al., 2019; Sconza et al., 2020). The absence of evidence and knowledge gaps still require a comprehensive review to enable healthcare decision makers to obtain accurate and credible summaries of the best available evidence on this topic.

The AMSTAR (a measurement tool to assess systematic reviews) was developed to evaluate the systematic reviews of randomized trials. It has been updated to AMSTAR2, which provides users a more detailed assessment of systematic reviews that include randomized or non-randomized studies of healthcare interventions. AMSTAR2 helps identify high-quality SRs and makes decision-making based on real-world observational evidence possible (Shea et al., 2007; Shea et al., 2017).

Umbrella reviews (URs) synthesize the results of the current body of multiple SRs and can provide a better quality evidence for clinical work on a topic of interest (Pollock et al., 2023). The purpose of this UR is to summarize the evidence from SRs on the efficacy and safety of ozone therapy in KOA.

## Materials and methods

The Preferred Reporting Items for Systematic reviews and Meta-Analyses (PRISMA) guideline and the tool AMSTAR2 were used to prepare the UR (Moher et al., 2009; Shea et al., 2017).

### Registration

The protocol was registered in the international prospective registry of systematic reviews (PROSPERO). Registration number: CRD 42019137746.

### Inclusion criteria

#### Participants

Adults aged 18 years and above of both sexes with a clinical or radiological diagnosis of KOA were included. Patients with other musculoskeletal diseases were excluded.

#### Interventions

IA, percutaneous, and/or systemic OT were administered for the treatment of KOA. No limitations regarding frequency or dose were applied.

#### Comparator

Placebo or any other types of pharmacological IA, percutaneous, and/or systemic therapies were used. Studies comparing OT with surgical treatment were excluded.

#### Outcomes

Quantitative measures of the impact of the disease on pain and functional disability were assessed by the visual analog scale (VAS), Western Ontario and McMaster Universities Osteoarthritis Index (WOMAC), Lequesne index, and others (Price et al., 1983; Lequesne et al., 1987; Bellamy et al., 1988).

#### Types of studies

SRs with or without meta-analysis of RCTs of treatments with OT for KOA published until December 2022 in any language were included. Considering that RCTs are more comparable to each other than non-randomized studies and that RCTs have better control of confounding than non-randomized studies, we chose to include only RCTs (Valentine and Thompson, 2013).

#### Timing and effect

For both outcomes of pain and function, we considered the follow-up times reported in the studies.

### Exclusion criteria

Narrative literature reviews, SRs of non-RCTs, and meta-analyses without SR were excluded.

### Search strategy

A literature search was carried out in the following databases: MEDLINE (via PubMed), Embase (via Elsevier), Cochrane Central, Virtual Health Library, and Dialnet. A search was also carried out in the gray literature database Open Gray and in Google Scholar. We searched references of studies read in full and contacted experts and study authors to identify additional SRs.

### Search strategy for MEDLINE via PubMed

[Osteoarthritis (MeSH Terms)] OR [Osteoarthritides (Text Word)] OR [Osteoarthrosis (Text Word)] OR [Osteoarthritis (Text Word)] OR; [Degenerative Arthritides; (Text Word)] OR [Osteoarthroses (Text Word)] OR; [Degenerative Arthritis; (Text Word)] OR [Arthroses (Text Word)] OR [Arthrosis (Text Word)] OR; [Osteoarthrosis deformans (Text Word)] AND [OZONE (Text Word)] OR; ozone injections; OR; ozone injection; OR; ozone therapy; OR [OZONE (MeSH Terms)].

The search strategies for the other bases are found in (Supplementary Table S1). To identify any non-published or updated review, we contacted the authors of recent SRs and one expert in OT in Brazil.

### Selection of studies

The relevant SRs were selected in two stages. In the first one, two review authors (VL/CAFA) independently screened the titles and abstracts identified during the search phase to identify the eligibility criteria. In the second stage, the same authors independently read the selected studies in full. A consensus meeting resolved any discrepancies. If the two authors did not reach a consensus, a third review author would act as the arbiter (NCPR).

### Data extraction

A data extraction form was previously prepared. After identifying the eligible SRs, the same two reviewers (VL/CAFA) independently extracted the following data:

General characteristics: language, country of origin, year of publication, funding, and setting.
Characteristics of participants: age and gender.
Intervention: the types of intervention and control with procedural information (duration and dose, as well as the route of administration).
Primary outcomes: pain and functional disability.
Secondary outcomes: rate of complications and adverse events.

### Methodological quality assessment

The same two authors independently evaluated the methodological quality of the included SRs using the instrument AMSTAR2 (Shea et al., 2017). The overall rating is based on the weakness of the following critical domains: Item 2) protocol registered before the commencement of the review; Item 4) adequacy of the literature search; Item 7) justification for excluding individual studies; Item 9) risk of bias from individual studies being included in the review; Item 11) appropriateness of meta-analytical methods; Item 13) consideration of the risk of bias when interpreting the results of the review; and Item 15) assessment of publication bias.

The overall confidence in the results of the UR was rated as follows:

High—0 or one non-critical weakness (NCW)—SR provides a precise and clear description of the results of the studies that deal with the question of interest.

Moderate—more than one NCW—the investigation has more than one weakness but no critical flaws. It may provide a precise and clear description of the results of the studies included in the review. Many NCW may reduce the confidence in SR. It may be suitable to move the classification down from moderate to low confidence.

Low—one critical flaw with or without NCW—SR has a critical flaw and may not provide a precise and clear description of the results of the studies included in the review.

Critically low—more than one critical flaw with or without NCW—the investigation has more than one critical flaw and should not be relied on to give a precise and clear description of the available studies (Shea et al., 2017).

### Strategy for data synthesis

There was a high degree of clinical heterogeneity (e.g., differences in the methods of application of OT related to concentration, volume of ozone, comparators, and the time of follow-up), which made quantitative synthesis impossible. We decided to report the data in a narrative synthesis, according to the following items: 1) the overall confidence in the results of SRs based on AMSTAR2; 2) the characteristics of included studies; 3) the many different primary studies included in the selected SRs; 4) the efficacy of OT; and 5) the main conclusions of the diverse SRs.

### Analysis of the degree of overlap in studies

An overlap in reviews results from the use of multiple identical primary studies in similar reviews and indicates the degree to which reviews address the same literature of primary research. To measure the level of publication overlap, the corrected covered area (CCA) has been introduced as a quantitative metric. In this methodological approach, researchers initiate the process by constructing a citation matrix that organizes primary publications in rows, and the different systematic reviews were included in the umbrella review in columns. Subsequently, determining the frequency with which a particular study is cited across systematic reviews becomes a straightforward calculation (Pieper et al., 2014).

The citation matrix was constructed in order to calculate the “corrected covered area” as follows:

CA=Nrc,

$$\text{CCA}=\left(N-r\right)/\left(\text{rc}-r\right),$$

CA=covered area; CCA=Corrected covered area.

N = number of included publications, including double counting. This is the sum of ticked boxes in the citation matrix.

r = number of rows (number of index publications).

c = number of columns (number of reviews).

The degree of overlap in studies was rated according to Pieper et al. (2014): CCA = 0–5: slight; 6–10: moderate; 11–15: high; and >15: very high.

### Quality assessment of included RCTs

In the eight SRs evaluated in this umbrella review, the quality assessment of included studies was analyzed by the authors of each SR. RCTs with a lower risk of bias could result in higher confidence in SR’s conclusions.

## Results

### Literature search

**FIGURE 1:**
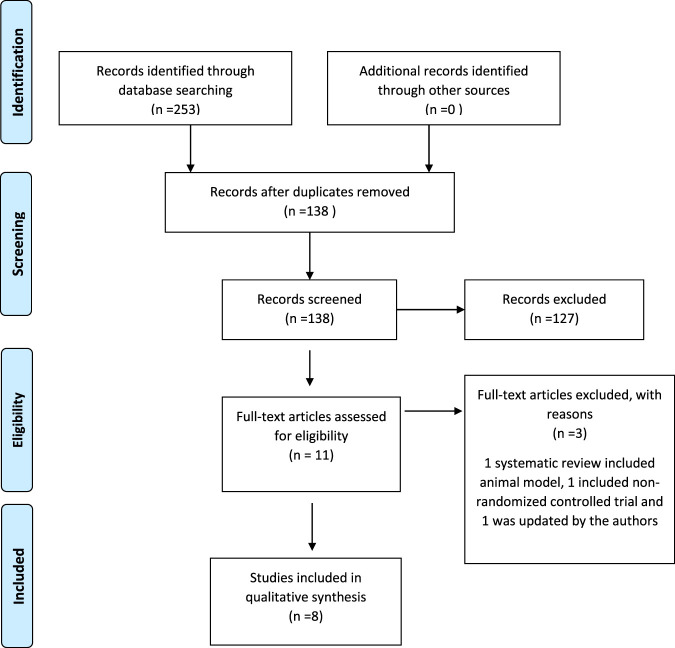
PRISMA flow diagram.

The literature search identified 253 records. After removing 115 duplicates, titles and abstracts of 138 records were independently screened and 127 were excluded. Eleven studies were read in full, and three were excluded (Anzolin and Bertol, 2018; Arias-Vázquez et al., 2019a; Noori-Zadeh et al., 2019) (Table 1). The two reviewers reached the consensus of all studies. One contact with two different authors was necessary (Li et al., 2018; Arias-Vázquez et al., 2019a). Eight studies were included in this research. Figure 1 shows the process according to the PRISMA flow diagram.

**TABLE 1:** Characteristics of included and excluded systematic reviews.

Included systematic reviews						
Author and year	Number of studies	Number of patients (I/C)	Intervention OMO dose, volume, and frequency	Comparator	Follow-up	Outcome
Zhu et al. (2015)	2	344 ozone group- 98	IA 10–15 mL and weekly four sessions (dose not mentioned)	Chinese herbal formulae	1 month	VAS, WOMAC, and Lysholm
Raeissadat et al. (2018b)	5	428 (225/203)	IA 15–30 mcg; 7–15 mL; and 3–8 weekly sessions	IAHD: three sessions weekly; IAHA: 1–4 weekly sessions. Air: eight weekly sessions of 10 mL of air	1 week to 12 months	VAS and WOMAC
Li et al. (2018)	4	298 (147/151)	IA 30–35 mcg; 10–20 mL; frequency not mentioned	IAHA 10 mg/2.0–2.5 mL	6–12 months	VAS and WOMAC
Costa et al. (2018)	6	494 (252/242)	IA 15–40 mcg; 5–15 mL; 3–5 weekly sessions periarticular points (5 mL at 10 μg/mL per point) 3 times during the 1st week, twice in the 2nd week, and once every next 3 weeks	IA HA: 20–40 mg/2 mL weekly- 5 sessions; with/without ozone; IAHD: 3 sessions weekly; radiofrequency of geniculated nerves; PRP: 2 monthly sessions	1 week to 12 months	VAS, WOMAC, and geriatric pain measure; KOOS; OKS; Lysholm; Lequesne; TUG; and short-form health survey
Arias-Vázquez et al. (2019a)	10	781 (400/381)	IA 15–30 mcg; 5–10 mL; frequency varied: only one session; once a month: 3 sessions/-weekly injections: 3 to 8 sessions; and −2 to 3 sessions/week	IA placebo: 8 sessions of 10 mL of air weekly; IA steroids: single session-40 mg methylprednisolone combined with exercise and paracetamol or ozone; IA HA: 1-5 weekly sessions with/without ozone; IAHD: 3 sessions weekly; and radiofrequency of geniculated nerves; PRP: 2 monthly sessions	3–6 months	VAS, WOMAC, OKS, Lequesne, levels of interleucin 1b, and TNF-alfa
Sconza et al. (2020)	11	858	IA 15–40 mcg 5–20 mL; 1–4 weekly sessions	IAHA, PRP, IAHD, radiofrequency, and corticosteroids- AINE	1–6 months	VAS, WOMAC, Geriatric Pain Measure, OKS, ROM Lysholm, and TUG EuroQoL
Oliviero et al. (2019)	6	353	IA 15–30 mcg; 6–10 mL: 3–8 weekly sessions	PRP, IAHA, placebo, IA HD, and corticosteroid	3–12 months	VAS, WOMAC, GPM, SF-36, and Lequesne
Javadi Hedayatabad et al. (2020)	2	129 (65/64)	3–4 weekly sessions (doses not mentioned)	IAHA	1–12 months	VAS and WOMAC
Excluded systematic reviews and justifications for exclusion						
Author and year	Justifications for exclusion					
Anzolin and Bertol (2018)	They included animal models studies					
Arias-Vázquez et al. (2019b)	The same group of authors published in the same year a systematic review with only eight RCTs (Arias-Vázquez et al., 2019b) and another with 10 RCTs (Arias-Vázquez et al., 2019a). We chose to include the second, more complete					
Noori-Zadeh et al. (2019)	They included non-randomized clinical trials					

C, control; HA, hyaluronic acid; HD, hypertonic glucose; I, intervention; IA, intra-articular; NRCT, non-randomized clinical trial; NSAID, non-steroidal anti-inflammatory drug; ODI, Oswestry Disability Index; OKS, Oxford knee score; OMO, oxygen therapy with medicinal ozone; PEDro, physiotherapy evidence database score (Serra et al., 2023); PRP, platelet-rich plasma; RCT, randomized controlled trial; RD, risk difference; ROM, range of movement; SC, subcutaneous; SMD, standard mean difference; WMD, weighted mean difference.

### Characteristics of included and excluded SRs

All SRs were published between 2015 and 2020. They analyzed a total of 15 RCTs that enrolled 3,685 patients. VAS and WOMAC scales were the preferred tools for the assessment of pain and/or function in all studies. The majority of SRs utilized the Kellgren–Lawrence grading system (grades 0–IV) for OA in their assessment (Raeissadat et al., 2018b; Costa et al., 2018; Li et al., 2018; Arias-Vázquez et al., 2019a; Oliviero et al., 2019; Sconza et al., 2020). As per this grading system, a significant portion of the studies focused on patients in the early or mid-stages of knee OA. The most frequent comparator was hyaluronic acid. The follow-up time for the outcome pain ranged from less than 1 month to 12 months. Most SRs included RCTs that applied the intervention to patients with mild to moderate stages of KOA and did not mention the settings of the studies. In relation to the strategy for data synthesis, more than half of the reviews performed quantitative synthesis. Table 1 presents the characteristics of included and excluded SRs. The results of quantitative synthesis and the safety of interventions are described in Supplementary Table S2.

### Methodological quality of SRs

No SR was excluded based on the methodological quality criteria. According to AMSTAR2, the overall confidence in the results of all eight studies was critically low. Only two SR established the review methods prior to conducting the review and registering the studies. Regarding the literature search, no SR performed all the steps to the comprehensive search. Furthermore, no SR provided a list of excluded studies. The critical appraisal results for each of the eight systematic reviews are summarized in Table 2.

**TABLE 2:** Assessment of the methodological quality of systematic review according to AMSTAR2 domains.

Author and year	Q1	Q2*	Q3	Q4*	Q5	Q6	Q7*	Q8	Q9*	Q10	Q11*	Q12	Q13*	Q14	Q15*	Q16	Overall confidence
Li et al. (2018)	Y	N	N	PY	Y	Y	N	PY	Y	N	N	N	N	N	N	Y	Critically low
Costa et al. (2018)	Y	Y	N	N	Y	Y	N	PY	Y	N	NA	NA	N	Y	NA	Y	Critically low
Arias-Vázquez et al. (2019a)	Y	N	N	N	Y	Y	N	PY	Y	N	NA	NA	Y	Y	NA	Y	Critically low
Raeissadat et al. (2018b)	Y	Y	N	N	Y	Y	N	Y	Y	N	Y	N	N	Y	N	Y	Critically low
Sconza et al. (2020)	N	N	N	N	Y	Y	N	PY	Y	N	NA	NA	Y	N	NA	Y	Critically low
Oliviero et al. (2019)	Y	N	N	PY	Y	Y	N	PY		N	N	N	N	N	N	Y	Critically low
Zhu et al. (2015)	Y	N	N	PY	Y	Y	N	PY	Y	N	Y	N	Y	Y	Y	Y	Critically low
Javadi Hedayatabad et al. (2020)	Y	N	N	PY		Y	N	PY	N	N	Y	N	Y	N	N	Y	Critically low

Legend: Y, yes; PY, partial yes; N, no; NA, not applicable; *, critical domains.

### Primary studies included in SRs

Six countries accounted for all the 15 RCTs: India, China, Iran, Italy, Turkey, and Brazil (Table 3). The citation matrix presents the level of overlapping in the reviews. Considering that N = 47 (number of included publications), r = 15 (number of index RCTs), and c = 8 (number of included SRs), the corrected covered area was 30.05, demonstrating a very high degree of overlap in this UR. Table 3 presents the citation matrix for SRs.

**TABLE 3:** Citation matrix for systematic reviews that assessed the effectiveness of oxygen therapy with medicinal ozone in KOA in clinical trials.

Systematic reviews/primary studies/ author, year/country [number of participants in each clinical trial]	Zhu et al. (2015)	Raeissadat et al. (2018b)	Li et al. (2018)	Costa et al. (2018)	Arias-Vázquez et al. (2019a)	Sconza et al. (2020)	Oliviero et al. (2019)	Javadi Hedayatabad et al. (2020)
Mishra, 2011/India [46]	—	—	—	—	X	X	—	—
Li, 2013/China [200]	X	—	—	—	—	—	—	—
Chen, 2013/China [144]	X	—	—	—	—	—	—	—
Momenzadeh, 2014/Iran [60]	—	—	—	—	—	—	—	X
Hashemi 2015/Iran [80]	—	X	—	X	X	X	X	—
Giombini 2016/Italy [46]	—	—	X	X	X	—	—	X
Chansoria 2016/India [80]	—	—	—	—	X	X	—	—
Hashemi, 2016/Iran [72]	—	—	—	—	X	X	—	—
Duymus, 2017/Turkey [102]	—	X	X	X	X	X	X	X
Feng, 2017/China [76]	—	—	—	X	—	X	—	—
Lópes de Jesus, 2017/Brazil [96]	—	X	—	X	X	X	X	—
Invernizzi, Italy/2017 [42]	—	X	X	—	X	X	—	X
Hashemi, 2017/Iran [61]	—	—	—	—	X	X	—	—
Raeissadat, 2018b/Iran [141]	—	X	X	—	X	X	X	X
Babaei-Ghazani, 2018/Iran [62]	—	—	—	—	—	X	X	—

Most SRs assessed the risk of bias of RCTs using the Cochrane risk-of-bias (RoB) tool for RCTs (Higgins et al., 2011). Only one SR did not present the assessment of the quality of the selected RCTs (Oliviero et al., 2019). The quality assessments are presented in Supplementary Table S3.

Considering all SRs included in this UR, we selected the more and the less rigorous evaluation of each study classified by RoB. At best, three RCTs showed a low risk of bias (Lopes de Jesus et al., 2017; Raeissadat et al., 2018a; Babaei-Ghazani et al., 2018). All the others presented a high risk of bias.

### Intervention characteristics

The frequency of interventions most commonly reported in RCTs was weekly injections, with three to eight sessions in total. The duration of treatments varied from 1 to 12 weeks. The volume of IA ozone and the concentration per dose varied from 5 to 20 mL and 15 to 40 mcg/mL, respectively. Periarticular injections were administered with 5–10 mcg/mL per anatomical point around the joint (Table 2).

### Efficacy and safety of OT for knee osteoarthritis

Three SRs reported statistically significant efficacy in groups treated with ozone, when compared to placebo for the outcome “pain reduction” (Raeissadat et al., 2018b; Costa et al., 2018; Arias-Vázquez et al., 2019a), but the intervention was not superior to other treatments in three reviews (Zhu et al., 2015; Li et al., 2018; Javadi Hedayatabad et al., 2020). Regarding function, only one investigation reported the efficacy of OT when compared to placebo (Arias-Vázquez et al., 2019a). One SR recommended OT as an efficient non-surgical treatment (Raeissadat et al., 2018b). All but one study examined safety, and OT was considered a safe procedure with a low incidence of mild adverse events reported. Table 4 presents the main conclusions and observations about the included SRs.

**TABLE 4:** Main conclusions of the included systematic reviews.

Author, year	Systematic reviews conclusion	Observation
Li et al. (2018)	“Intra-articular injection of hyaluronic acid was associated with a significant reduction in the VAS score at the first month compared to O_2_–O_3_. There were significant differences in WOMAC stiffness and function at a 6-month follow-up between groups. Based on the current evidence available, more RCTs are needed for further investigation of efficacy of ozone therapy for the treatment of KOA”	Data mentioned in the text of the paper are different from data shown in the forest plots in relation to VAS but did not alter the effects
Costa et al. (2018)	“Ozone therapy has proven efficacy in pain control and improvement of function in KOA in the short term in relation to the placebo when used in combination with hyaluronic acid, but it was not superior to other treatments. More randomized studies are needed to evaluate the risks/benefits of ozone therapy for this condition, both in the short term and the medium/long term”	—
Arias-Vázquez et al. (2019a)	“Intra-articular ozone infiltration appears to be an effective therapeutic intervention for KOA in the short term. However, studies with better methodological quality are needed to confirm its efficacy and to analyze long-term safety”	This systematic review included one RCT that applied ozone both in the intervention and in the controlled group (ozone x ozone with corticosteroids) (Borrelli et al., 2015). Another included RCT-associated local heat, paracetamol, and exercise both in the intervention and in the control group (Lopes de Jesus et al., 2017). It was not possible to draw conclusions about the effectiveness of *ozone* in these cases
Raeissadat et al. (2018b)	“Intra-articular ozone injection efficacy was significantly superior to placebo and slightly lower to other control injections with non-significant difference. Therefore, ozone could be recommended as an efficient non-surgical treatment, durable for at least 3–6 months, in mild or moderate knee OA management”	When authors performed sensitivity analysis after removing RCT that controlled the ozone group with placebo, the effects favoring ozone at first and third months have disappeared
Sconza et al. (2020)	“The included RCTs had poor methodological quality. Most of these studies were flawed by relevant bias, limiting conclusions on the efficacy of ozone therapy for KOA compared with other treatments. However, ozone proved to be a safe approach for pain control and functional recovery in the short-middle term management of KOA”	This systematic review included one RCT that applied *ozone* in the intervention and in the group controlled with corticosteroids Borrelli et al. (2015)
Oliviero et al. (2019)	“The weakness of included RCTs limits conclusion. Ozone injections can reduce pain, but there is no evidence that they slow down the evolution of disease in the long term. Ozone can be used as a conservative therapeutic option in the short-term management of KOA”	Heterogeneity not analyzed
Zhu et al. (2015)	“No significant differences were observed when Chinese herbal formulae were compared with intra-articular ozone therapy for KOA treatment. The conclusions were limited due to the poor methodological quality of included trials”	—
Javadi Hedayatabad et al. (2020)	“There was no significant difference between hyaluronic acid and ozone in reducing pain and improving function in patients with KOA, although the overall results favored hyaluronic acid over ozone”	This systematic review included one RCT that compared O_2_ (not ozone) with hyaluronic acid for management of KOA

HA, hyaluronic acid; KOA, knee osteoarthritis; RCT, randomized controlled trial; SR, systematic review; VAS, visual analog scale.

## Discussion

### Main findings

Umbrella reviews are employed when there are many SRs on the same subject, aiming to summarize their results and support clinical and health planning decisions (Pollock et al., 2023). This UR synthesized the available evidence on the efficacy of OT in relieving KOA pain and function improvement. It was based on a thorough literature search and assessment of study quality. All eight SRs presented critically low methodological quality according to AMSTAR2 (Shea et al., 2017). As most SRs included in this UR did not present a protocol prior to conducting the review, we highlight the need for systematic reviews’ authors to pay more attention to this process.

None of the SRs met all the requirements of a comprehensive bibliographic search. Considering that OT is practiced in more than 40 countries worldwide, if the researcher does not search studies in different languages and in the gray literature, the SR may omit important research in that area (Quintero and Schwartz, 2012).

No SR presented a list of potentially relevant RCTs with justification for the exclusion of each one. Reasons to exclude a study are related to inappropriate population, intervention, or control. Sufficient knowledge on the study characteristics can help readers decide whether the study should be selected as unjustified exclusion may bias the review findings (Shea et al., 2017).

Different problems were identified in SRs that performed quantitative synthesis, including the absence of analysis of the investigation of risk of bias, (Raeissadat et al., 2018b; Li et al., 2018; Oliviero et al., 2019; Javadi Hedayatabad et al., 2020), heterogeneity in RCTs, (Oliviero et al., 2019), and the use of the same substance in both arms of the RCTs (Arias-Vázquez et al., 2019a). In addition, the level of overlapping in studies across SRs was very high, meaning that many RCTs appeared several times, leading to unintentionally stronger weighting in any meta-analyses (Pieper et al., 2014).

Based on the RoB assessment, the majority of RCTs examined in the SRs utilized in this UR displayed a substantial risk of bias. Among those that did meet the predefined quality criteria, their findings can serve as a foundation for informing and shaping the design of upcoming studies (Lopes de Jesus et al., 2017; Raeissadat et al., 2018a; Babaei-Ghazani et al., 2018).

Despite the previously mentioned aspects that limit the quality of SRs, no review was excluded based on the criteria of methodological quality. It is important not to interpret low-quality evidence as the evidence of no effect. Low-quality evidence means unclear evidence, and findings should stimulate more research (Jamtvedt et al., 2008). Most weaknesses resulting from flaws in the methodology of SR could be avoided if the authors used a tool like AMSTAR or AMSTAR2 before starting the investigation.

### Efficacy and safety of OT

Despite SRs highlighting methodological limitations in RCTs, the majority conclude that OT enhances short-term (3–6 months) pain management in mild-to-moderate KOA (Raeissadat et al., 2018b; Costa et al., 2018; Li et al., 2018; Arias-Vázquez et al., 2019a; Oliviero et al., 2019). In these instances, OT appears superior to the placebo but not surpassing the intra-articular injections of hyaluronic acid (Raeissadat et al., 2018b; Li et al., 2018; Javadi Hedayatabad et al., 2020). Results concerning the improvement of physical function are less consistent. Ozone therapy reduces the release of proteolytic enzymes and pro-inflammatory cytokines, leading to a decrease in intra-articular edema. Additionally, it stimulates the synthesis of chondrocytes and fibroblasts (Raeissadat et al., 2018a), demonstrating biological plausibility for modifying the disease course. OT has proven to be a safe procedure (Arias-Vázquez et al., 2019a), with no serious adverse events reported in any of the SRs in this study. A recent evidence map on OT has similarly revealed no serious adverse effects (Serra et al., 2023). In summary, these findings suggest that intra-articular OT could be considered a safe and effective short- to mid-term treatment for KOA patients. However, further high-quality studies are needed to enhance the scientific understanding of this promising conservative intervention.

### Strengths and limitations

This study has some positive aspects. To the best of our knowledge, it is the first attempt to review the existing evidence of ozone therapy in KOA using a standardized methodology for an overview. The protocol was pre-specified, and the variables of interest were clearly defined. Methodological quality was evaluated using AMSTAR2 as a guiding framework, which is known for its reliability, construct validity, and feasibility. Furthermore, the assessment of the amount of overlapping in SRs contributed to the improvement of the methodological quality of this UR.

This study has a number of limitations. Our focus was restricted to systematic reviews, and we did not reference primary research articles. Consequently, our synthesis was constrained to the authors’ interpretation of the primary data encompassed in the review. Like any umbrella review, definitive conclusions regarding the sources of heterogeneity and other potential biases cannot be firmly established. Lastly, while the majority of the included reviews were recently published, it is crucial to acknowledge that reviews may become outdated swiftly due to the rapid emergence of new research. Therefore, insights should be interpreted in the context of this dynamic research landscape.

## Conclusion

This umbrella review highlights the potential positive impact of OT on pain management in KOA. OT is promising as KOA treatment, due to its safety and relative ease of administration. Ensuring proper adherence to guidelines such as PRISMA and AMSTAR2 has the potential to enhance the quality of SRs in this context.
