# Supplementary material for: Efficacy and safety of ozone therapy for knee osteoarthritis: an umbrella review of systematic reviews
Source: Front Physiol. 2024 Feb 20;15:1348028. doi: 10.3389/fphys.2024.1348028 (PMC10912569; doi:10.3389/fphys.2024.1348028)
Supplement: Supplementary file 1 [file Table1.DOCX]

S1 Table. Search Strategy

| Base | Total |
| --- | --- |
| EMBASE  ('ozone therapy'/exp OR 'ozone therapy') AND ('osteoarthritis'/exp OR 'osteoarthritis') | 65 |
| Virtual Health Library  "Osteoarthritis" AND “ozone therapy” | 30 |
| Cochrane CENTRAL  osteoarthritis OR osteoarthritides OR osteoarthrosis OR osteoarthroses OR "degenerative arthritides" OR "degenerative arthritis" OR arthroses OR arthrosis (Word variations have been searched)  #2 MeSH descriptor: [Osteoarthritis] explode all trees  #3 Ozone therapy  #4 #1 OR #2  #5 #3 AND #4  Filter- Systematic review | 3 |
| Google Scholar  Osteoarthritis" AND “ozone therapy  The firs 100 citations | 1 |
| DIALNET  Osteoarthritis+"ozone+therapy" | 7 |
| Open Grey  Ozone therapy Osteoarthritis | 0 |
|  | |
