# Supplementary material for: Efficacy and safety of ozone therapy for knee osteoarthritis: an umbrella review of systematic reviews
Source: Front Physiol. 2024 Feb 20;15:1348028. doi: 10.3389/fphys.2024.1348028 (PMC10912569; doi:10.3389/fphys.2024.1348028)
Supplement: Supplementary file 2 [file Table3.DOC]

Table S3A. Risk of bias in RCT according to Cochrane’s RoB tool considering the more rigorous evaluation and the worst classification of at least one Systematic Review

|  | Duymus 2017 | Giombini (2016) | Invernizzi (2017) | Raeissadat 2018 | Hashemi 2015 | Feng  (2017) | Jesus  (2017) | Mishra (2011) | Hashemi (2016) | Hashemi (2017) | Chansoria(2016) | Ghazani (2018)  * | Chen (2013)  * | Li, (2013)  * |
| --- | --- | --- | --- | --- | --- | --- | --- | --- | --- | --- | --- | --- | --- | --- |
| **Random sequence generation (selection bias)** | **+** | **+** | **?** | **?** | **?** | **+** | **+** | **?** | **?** | **+** | **-** | **+** | **+** | **+** |
| **Allocation concealment (selection bias)** | **?** | **?** | **?** | **?** | **?** | **?** | **+** | **?** | **?** | **?** | **?** | **+** | **?** | **?** |
| **Blinding of participants and personnel (performance bias)** | **-** | **?** | **-** | **?** | **-** | **-** | **+** | **?** | **-** | **-** | **-** | **+** | **?** | **?** |
| **Blinding of outcome assessment (detection bias)** | **-** | **?** | **?** | **?** | **-** | **-** | **?** | **?** | **-** | **+** | **-** | **+** | **?** | **?** |
| **Incomplete outcome data** | **-** | **-** | **-** | **-** | **?** | **?** | **+** | **-** | **?** |  |  | **+** | **+** | **+** |
| **Selective reporting (reporting bias)** | **?** | **?** | **-** | **?** | **?** | **?** | **+** | **-** | **-** | **-** | **+** | **+** | **?** | **?** |
| **Other bias** | **?** | **?** | **-** | **?** | **-** | **?** | **?** | **-** | **?** | **?** | **-** | **?** | **?** | **?** |
|  | **High risk bias** | **High risk bias** | **High risk bias** | **High risk bias** | **High risk bias** | **High risk bias** | **Moderate risk bias** | **High risk bias** | **High risk bias** | **High risk bias** | **High risk bias** | **Moderate risk bias** | **High risk bias** | **High risk bias** |

*Only one systematic review evaluated this RCT

+ Low risk; ? unclear risk; - High risk;

Table S3B. Risk of bias in RCT according to Cochrane’s RoB tool considering the less rigorous evaluation and the best classification of at least one systematic review

|  | Duymus (2017) | Giombini  (2016) | Invernizzi (2017) | Raeissadat (2018) | Hashemi (2015) | Feng (2017) | Jesus (2017) | Mishra (2011) | Hashemi (2016) | Hashemi (2017) | Chansoria  (2016) | Ghazani (2018)  * | Chen (2013) | Li, (2013) |
| --- | --- | --- | --- | --- | --- | --- | --- | --- | --- | --- | --- | --- | --- | --- |
| **Random sequence generation (selection bias)** | **+** | **+** | **+** | **+** | **?** | **+** | **+** | **?** | **?** | **+** | **+** | **+** | **+** | **+** |
| **Allocation concealment (selection bias)** | **?** | **+** | **+** | **+** | **?** |  | **+** | **?** | **?** | **?** | **?** | **+** | **?** | **?** |
| **Blinding of participants and personnel (performance bias)** | **+** | **+** | **-** | **+** | **?** | **?** | **+** | **?** | **?** | **+** | **?** | **+** | **?** | **?** |
| **Blinding of outcome assessment (detection bias)** | **?** | **?** | **+** | **+** | **?** | **?** | **+** | **?** | **?** | **+** | **?** | **+** | **?** | **?** |
| **Incomplete outcome data** | **+** | **+** | **+** | **+** | **?** | **?** | **+** | **?** | **?** | **?** | **?** | **+** | **+** | **+** |
| **Selective reporting (reporting bias)** | **+** | **?** | **?** | **+** | **+** | **+** | **+** | **?** | **?** | **?** | **+** | **+** | **?** | **?** |
| **Other bias** | **+** | **+** | **+** | **+** | **+** | **+** | **+** | **+** | **+** | **+** | **+** | **?** | **?** | **?** |
|  | **Moderat risk bias** | **Moderat risk bias** | **Moderat risk bias** | **Low risk bias** | **High risk bias** | **High risk bias** | **Low risk bias** | **High risk bias** | **High risk bias** | **High risk bias** | **High risk bias** | **Low risk bias** | **High risk bias** | **High risk bias** |

Table S3C- Quality assessment of the included studies using the Jadad Scale by Hedayatabad

| Study | Was the study described as randomized?* | Was the study described as a double blind?* | Was there a description of withdrawal and dropouts?* | The randomization scheme described and appropriate* | The method of double blinding described and appropriate* | The randomization scheme described and inappropriate** | The method of double blinding described and inappropriate** | Total score |
| --- | --- | --- | --- | --- | --- | --- | --- | --- |
| Raeissadat (2018) | 1 | 1 | 1 | 1 | 1 | 0 | 0 | 5 |
| Duymus (2017) | 1 | 0 | 1 | 1 | 0 | 0 | 0 | 3 |
| Giombini (2016) | 1 | 0 | 0 | 1 | 0 | 0 | 0 | 2 |
| Invernizzi (2017) | 1 | 0 | 1 | 1 | 0 | 0 | 0 | 3 |
| Momenzadeh (2014) | 1 | 1 | 1 | 0 | 0 | 0 | 0 | 3 |

*Yes: +1; no: 0/ **Yes: -1; no: 0. Jadad score ranges from 0 to 5. Scores of 3 or higher is considered appropriate^55^.
