# Supplementary material for: Efficacy and safety of ozone therapy for knee osteoarthritis: an umbrella review of systematic reviews
Source: Front Physiol. 2024 Feb 20;15:1348028. doi: 10.3389/fphys.2024.1348028 (PMC10912569; doi:10.3389/fphys.2024.1348028)
Supplement: Supplementary file 3 [file Table2.DOCX]

Table S2. Characteristics of included systematic reviews: quantitative synthesis and safety

| Author,year, | Quantitative synthesis | Safety |
| --- | --- | --- |
| Li, 2018^21^ | ***Pain***  1^st^ month  VAS  WMD= -0.463 (95% CI -0.85, -0.06)  3^rd^ month  VAS  WMD = -0.95 (95% CI -2.420, 0.51)  6^th^ month  VAS  WMD=0.884 (95% CI -2.407, 0.639)  WOMAC  WMD= -0.359 (95% CI -1.16, 0.44)  ***Function***  (period not mentioned)  WOMAC  WMD= -4.748 (95% CI -9.21, -0.28) | Incidence rate of adverse effects  RD= 0.006 (95% CI:-0.047, 0.058 p=0.837) |
| Raeissadat, 2018^19^ | 1^st^ month  VAS  MD = −0.23  (95% CI –1.46, 1.00)  2-3^rd^ month  VAS  MD =0.28 (95% CI  –1.46, 2.02)  4-6^th^  VAS  MD =1.31 (95% CI  –2.02, 4.64)  12^th^ month  VAS  MD =0.80 (95% CI 0.43, 1.17) | Adverse events were similar to both groups. None have reported any major complications |
| Oliviero, 2019^18^ | 1^st^ month  VAS  MD = −0.05  (95% CI –0,3, 0.2)  WOMAC  MD= 5.33 (95% CI 2.53, 8.2)  3^rd^ month  VAS  MD = -0.4 (95% CI  –0.61, -0.19)  WOMAC  MD= -3.95 (95% CI -6.11, -1.79)  6^th^month  VAS  MD =1.37 (95% CI 20.5, 22.2)  WOMAC  MD= 21.4 (95% CI 2.53, 8.2)  12^th^ month  VAS  MD = 1.65 (95% CI 1.43, 1.87)  WOMAC  MD= 15.8 (95% CI 14.09, 17.51) | No severe adverse events were recorded |
| Zhu, 2015^39^ | VAS  MD= 0.05 (95% CI -0.37, 0.47)  WOMAC  SMD= -0.34 (95% CI -0.73, 0.06)  Lysholm  MD= -0.50 (95% CI -3.54, 2.54) | No severe adverse events were recorded |
| Hedayatabad, 2020^36^ | VAS  SMD  1 month  1.822 (95% CI 0.002, - 3.641)  2-6 months  1.549 (95% CI -0.846, - 3.944)  >6 months  0.193 (95% CI -4.562, - 4.948) | The systematic review did not investigate adverse events. |
|  |  |  |

MD- mean difference; OMO- Oxygen therapy with medicinal ozone; RD- risk difference;SMD- standard mean difference; VAS- Visual Analogue Scale; WMD- weighted mean difference
